# Forecasting the Romanian Unemployment Rate in Time of Health Crisis—A Univariate vs. Multivariate Time Series Approach

**DOI:** 10.3390/ijerph182111165

**Published:** 2021-10-24

**Authors:** Adriana AnaMaria Davidescu, Simona-Andreea Apostu, Aurel Marin

**Affiliations:** 1 Department of Statistics and Econometrics, Bucharest University of Economic Studies, 010552 Bucharest, Romania; simona.apostu@csie.ase.ro; 2 Department of Education, Training and Labour Market, National Scientific, Research Institute for Labour and Social Protection, 010643 Bucharest, Romania; 3 Institute of National Economy, 050711 Bucharest, Romania; 4 Department of Physical Education and Sports, National University of Physical Education and Sports, 060057 Bucharest, Romania; aurel.marin@unefs.ro

**Keywords:** unemployment rate, bibliometric analysis, ARFIMA, SETAR, VAR, VECM, cointegration, Romania

## Abstract

Economic crises cause significant shortages in disposable income and a sharp decline in the living conditions, affecting healthcare sector, hitting the profitability and sustainability of companies leading to raises in unemployment. At micro level, these sharp decreases in earnings associated with unemployment and furthermore with the lack of social protection will impact the quality of life and finally the health of individuals. In time of crisis, it becomes vital to support not only the critical sectors of the economy, the assets, technology, and infrastructure, but to protect jobs and workers. This health crisis has hit hard the jobs dynamics through unemployment and underemployment, the quality of work (through wages, or access to social protection), and through the effects on specific groups, with a higher degree of vulnerability to unfavorable labor market outcomes. In this context, providing forecasts as recent as possible for the unemployment rate, a core indicator of the Romanian labor market that could include the effects of the market shocks it becomes fundamental. Thus, the paper aims to offer valuable forecasts for the Romanian unemployment rate using univariate vs. multivariate time series models for the period 2021–2022, highlighting the main patterns of evolution. Based on the univariate time series models, the paper predict the future values of unemployment rate based on its own past using self-forecasting and implementing ARFIMA and SETAR models using monthly data for the period January 2000–April 2021. From the perspective of multivariate time series models, the paper uses VAR/VECM models, analyzing the temporal interdependencies between variables using quarterly data for the period 2000Q1–2020Q4. The empirical results pointed out that both SETAR and VECM provide very similar results in terms of accuracy replicating very well the pre-pandemic period, 2018Q2–2020Q1, reaching the value of 4.1% at the beginning of 2020, with a decreasing trend reaching the value of 3.9%, respectively, 3.6% at the end of 2022.

## 1. Introduction

Economic crises cause significant shortages in the disposable income and a sharp decline in the living conditions, affecting healthcare sector, hitting the profitability and sustainability of companies leading to raises in unemployment. At micro level, these sharp decreases in earnings associated with unemployment and furthermore with the lack of social protection will impact the quality of life and finally the health of individuals.

The crisis caused by the coronavirus impacted the entire planet. The London Business School report from March 2020 [1] indicated that the COVID-19 pandemic is the worst health crisis of the modern era and, even if kept under control, will cause a major global economic crisis. 

Beyond morbidity and mortality, the pandemic affected the world economies in different pervasive sectors, such as travel, tourism, supply chains, stock market instability, and oil price fluctuations [2], imports and exports, pressure on the health sector being the most important element to control worldwide.

In order to fight with the new virus, were taken massive lockdown measures, conducing to slowing down economic activity, as the production units have closed down. As a result, we registered a massive unemployment rate in history. 

The EU economy contracted by 6.1% and the Eurozone economy by 6.6% in 2020. Although businesses and consumers have generally adapted to better cope with isolation measures, some sectors, such as tourism and services provided in person, continue to suffer. Europe’s economies began to recover last summer, but in the fourth quarter of 2020 and the first quarter of 2021 stagnated, due to public health measures introduced in order to limit the increase on COVID-19 cases. The EU and Eurozone economies are expected to recover sharply as vaccination rates rise and restrictions are relaxed. This growth will be driven by private consumption, investment, and increased demand for EU exports from a strengthened global economy. Labor market conditions are slowly improving after the initial impact of the pandemic. Employment increased in the second half of 2020 and unemployment rates fell below their maximum levels in most member states [3].

Labor market indicators, such as labor force participation, employment, unemployment, and the unemployment rate are key indicators of national, regional, and local economic conditions. With their help, funds are allocated, the local labor supply is monitored, so their forecast is of great interest, especially in crisis conditions [4]. 

In Romania, the coronavirus crisis affected activities in many sectors, the number of unemployed increased. In addition, many unemployed emigrants returned to the country, making the situation even more difficult. In the period July–August 2021 the unemployment rate reached a maximum of 5.3% and fell slightly, but remained above 5% in September. 

Unemployment affected also the health system; people no longer having a place to work and having lost their health insurance. As Romania had one of the weakest medical systems in the European Union, the shortcomings of this system and unemployment made the fight against the new virus very difficult.

Therefore, in this specific context, the paper aims to offer valuable forecasts for the Romanian unemployment rate an important indicator of labor market strongly affected by the recent pandemic crisis, using both univariate methods of forecasting (ARFIMA and SETAR models) and multivariate models (VAR or VECM models), in order to monitor and to put under control a core indicator which is part of the economy’s strategy in times of pandemic. For this, we have used monthly data for univariate methods covering the period January 2000–April 2021, and quarterly data for multivariate techniques, covering the period 2000Q1–2020Q4, both of them divided into training data covering the period January 2000–June 2018 and test data covering the last three years, 2018–2020, with the unemployment out-of-sample predictions on the next two years, 2021–2022. The study exploits modeling unemployment rate determining the goodness of fit, as well as the validity of the assumptions and selecting an appropriate and more parsimonious model thereby proffer useful suggestions and recommendations. 

Therefore, our research focused on the following research questions: Is the unemployment rate expected to decrease in the period 2021–2022? Do the multivariate models offer better performance in forecasting the unemployment rate in comparison with univariate models? 

Thus, the core hypothesis of the paper is the following:

**Hypothesis** **1** **(H1).** *The unemployment rate is decreasing during the period 2021–2022*.

The paper is organized as follows. The section of literature review presents an overview of the most important studies regarding this topic of forecasting unemployment rate, while Section 3 explores the implications between unemployment and healthcare services in the context of economic crisis using the bibliometric analysis based on the most relevant studies from the field. Section 4 was dedicated to the presentation of univariate (ARFIMA and SETAR) vs. multivariate methods (VAR/VECM). Section 5 incorporates information related to the data used in the analysis and the main empirical results of all three methods. The paper provides also a section of forecasting performance comparison analyzing the forecast accuracy of univariate vs. multivariate models for out of the sample datasets. The paper ends with the main conclusions.

## 2. Literature Review

Public health research areas are following the economy, unemployment being a typical example [5]. If individual level studies show that unemployment is associated with a poorer state of health, both physically and psychologically [6], at the macroeconomic level studies are limited, few studies analyzing the phenomenon of economic crisis in the meaning of a sharp transition to a recession [7]. 

Unemployment appears when supply exceeds demand regarding labor, being considered a resource measured for a period of time. The unemployed are people who want to work, but do not have a job with wages available on the market [8]. The unemployment rate is characteristic for labor market, representing the percentage of the labor force that is unemployed, but registered as a jobseeker [9]. 

Unemployment is distributed differently across certain population subgroups, with the highest rates among black youths, followed by young people and ethnic minorities [10].

An economic crisis implies a deep, structural, and multi-faceted economic crisis, the result being a large fiscal deficit, huge public debt, and the continuous erosion of the country’s competitive position [11]. Other negative effects of the economic crisis can also be observed at social level, affecting employment, and challenging or increasing unemployment rate. Economic crises are associated with lower labor demand, disposable income reduction, problems on health financing and deterioration of access to healthcare [12].

The crisis is significantly affecting a country’s economy, the consequences being an increase in poverty and social inequality [13], health system deficiencies, and indicators that reflect the population health status [14]. Economic crisis affects mental health differently depending on the employment situation and educational level [15].

The worst consequences of the crisis in socio-economic life are represented by: unemployment, job insecurity, reduced incomes, poverty, and increased mental disorders. The reduction in government spending affects the structure and operation of public hospitals, which face understaffing, a shortage of medicines, and basic medical supplies. Thus, along with severe austerity measures, the health system [16] is severely affected.

In developed countries the macro-economic situation significantly influences population health conditions [17,18], but there are also studies according to which the well-being of the population is not very much influenced by the macroeconomic situation. In addition, some studies have shown that also a developing economy leads to good results on health outcomes.

During economic crises, the demand for healthcare services and the utilization of such services follows the general drop in socioeconomic status [19,20,21], reflecting barriers to access due to increased unemployment and reductions in disposable income [22].

The crisis is exacerbating job insecurity, especially for employees in industries affected by many redundancies [23]. Reducing the number of employees leads to stressful conditions at work, due to increased workload, longer shifts and shorter breaks, reduced wages, and job dissatisfaction [24]. These lead to increased stress at work and, implicitly, to development of mental illness [25,26,27,28,29], in some cases representing a risk factor of suicide [30]. 

During the economic recession, mortality rates fall, especially for the elderly. Increasing the state unemployment rate involves improving physical health, reducing unhealthy behaviors and the use of health care [31]. The 2007–2009 economic crisis in the United States led to the slowest annual growth rate of health spending [13], only 3.9%, with hospital services stagnating at 4.9% [32]. The result of an economic crisis is also the use of medical care, during periods of economic contraction registering decreases generated by the decrease in the demand for medical care [33,34].

In developed countries studies have shown that unemployment rate is associated with health status, while in developing countries, socio-economic change influences to a greater extent the improvement of health [35]. Thus, Wang [36] showed that the unemployment rate is positively associated with mortality, an increased unemployment rate being unfavorable to the health outcomes of the population.

Unemployment rate represents probably the most important consequence of a recession [18]. The unemployment rate is rising rapidly in response to an economy heading in the wrong direction, changing the composition of the unemployed population. Thus, redundancies are resorted to, including healthy and productive employees, the unemployed can be both people with higher education and people with lower education, both people in good health and people with health problems [18].

Poor health is strongly correlated with high unemployment, being the result of selective processes [37], and deterioration of health due to unemployment [38]. This association is influenced by the economic situation of a country: people in poor health try to integrate into the labor market in the post-crisis periods [39,40].

Unemployment negatively influences health [6,41], this relation being a reciprocal one, the selection on health, registering simultaneous with the effects of unemployment on health [42], becoming unemployed involves stress that can deteriorate health [40,43].

During the crisis unemployment is more prevalent, being more harmful to health, including mental health during periods of high unemployment. As unemployment affects women more, action should be taken to reduce unemployment and support employment for women [44].

Heggebø [37] showed that redundancies target people with health problems more than healthy people, and in some cases younger people, being different depending on the country. In addition to the impact of crises on the overall health of individuals, some studies have looked at the impact of the crisis on health care, showing that reduced public spending on health care and reduced public hospital budgets due to the recession have led to organizational problems [12].

The increase in unemployment rates leads to the treatment by doctors of a smaller number of privately insured patients, both in hospital and outpatient. In contrast, physicians who record loss of income due to crises provide more care to patients with basic insurance as the unemployment rate rises [45]. Other effects regarding healthcare in case of crisis are: increases in the use of publicly funded health care services and NGOs facilities, reductions in public health spending, increasing self-reported unmet needs for examinations, increased demand of emergency services, reductions in coverage [46].

At European level, the results of studies have shown that unemployment is medicalized, to a greater or lesser extent, varying substantially from one country to another, depending on the level of unemployment and medical generosity [11].

Overall, the economic situation significantly impacts living conditions, with healthcare being severely affected. Political decision-makers should not neglect the implications that austerity and fiscal policies have on the health sector, and it is necessary to ensure that people continue to receive public healthcare and have access to preventive and social care services. This requires human-centered approaches, protecting human dignity and moral values [16].

The coronavirus crisis in 2020 has caused health problems, and destabilized the global economy. Mitigation measures have led to home orders, the closure of certain businesses, reduced demand for products and services, so that many workers have stopped working. One of the consequences was the increase in initial unemployment insurance claims, with great uncertainty about jobs and the companies that will remain in operation at the time [47].

Strong austerity measures have had a significant effect on the global economy, leading to an increase in the global unemployment rate [48], with long-term unemployment being a major challenge for society. As periods of unemployment lengthen, individuals deplete their savings [49,50], deteriorating and threatening productivity even after the end of the crisis [51].

Su et al. [52] analyzed the impact of COVID-19 on unemployment in European countries, highlighting that unemployment increased due to pandemic, slowing the economic activities and causing ceasing the industry and service sectors production activities. These led to rapid public policy all over the world in order to reduce the impact of mass unemployment, changing the community perceptions on individuals who are out of work and rely on government income support [53].

Unemployment caused by coronavirus could conduce to significant health loss, increase health inequities from, implying additional health system costs in high-income country. Therefore, prevention measures should be considered in order to reduce this risk, adding additional job creation programs and measures directed towards cardiovascular disease prevention [54,55]. 

The pandemic influenced employment differently. For example, in UK employment for young people aged 16–24 decreased by 6%, for older workers aged 65+ decreased by 8% and for those aged 25–64 decreased by 1.1% [56]. In the US, unemployment registered higher values among people experiencing long-standing health disparities, such as Hispanic and Black individuals, people with lower income, and lesbian, gay, bisexual, transgender, or nonbinary individuals [57]. Additionally, high figures of unemployment were indicated in case of migrant workers, contract workers and day laborers, facing unique risks due to the nature of their jobs, deepening the existing problems [58].

For the EU27, unemployment rate increased by less than 1.1%, instead in the US has undergone many changes. From January to April the US unemployment increased by more than 10 percentage points, falling by 7% in November [59]. In Ireland unemployment rate, increases to 18.5% of the total labor force, as consequence of decreasing labor demand and labor force participation [60].

## 3. Exploring the Implications between Unemployment and Healthcare Services in the Context of Economic Crisis—A Bibliometric Analysis

Bibliometric analysis is an integrative investigation tool of the literature, in a systemic and systematic process, on the vectors of interconnected topics, which allows by structuring and ordering the results obtained to convert quantitative to qualitative, by crystallizing concepts, identifying inter conditionalities, detecting trends, in order to increase the efficiency of research and decision makers’ access to conclusive and relevant information.

Therefore, the purpose of this demarche was to analyze the most relevant studies in the field using bibliometric analysis, the main source of scientific articles analyzing the implications of economic crisis on unemployment and healthcare services being the academic platform Google Scholar. In order to improve the selection of our scientific publications, we identified and used the following keywords: “economic crisis”, “unemployment”, and “health” in order to capture the relationship between the concepts. Therefore, we have explored the full content of 28 research articles, in order to highlight the structure of the scientific field, using the content analysis who inspects the most common words, the relationship between words: bi-grams and correlations in the analysis of this cross-cutting topic.

In order to identify the main topic of a document, an important measure is the word clouds giving the words with the highest frequency. Additionally, the relationships between words can be explored investigating which words tend to follow others immediately, or that tend to co-occur within the same documents. Both types of analyses are complementary. If the word network reveals which are the word pairs that co-occur most often, the correlation network reveal which words appear more often. Analyzing the network of co-occurrences, co-occurrences with a frequency of at least 20 times have been taken into account, with a correlation degree greater than 0.5. The analysis has been performed in R using the following libraries: tm, tidytext, quanteda, tidyverse, corpus, textmineR, tidyr, Rweka, wordcloud2, igraph, ggraph, widyr, stats, ldatuning, stm, readr, readtext, reshape2, ape, and dendextend.

**Figure 1:**
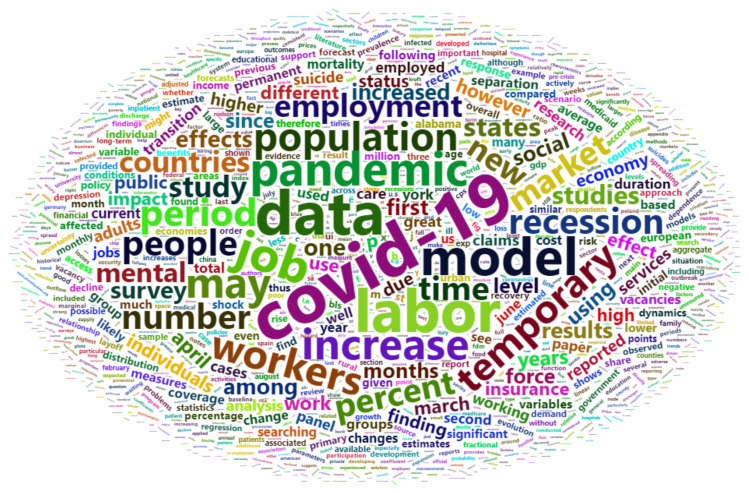
Most common words in selected scientific publications.

**Figure 2:**
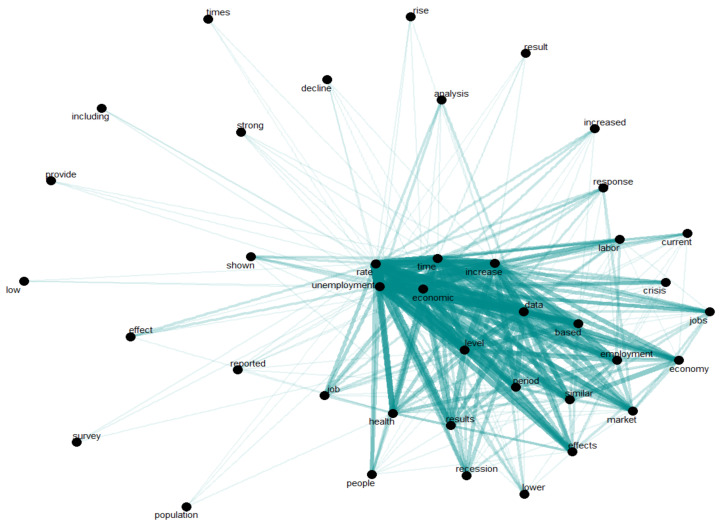
Word network in scientific publications’ content.

**Figure 3:**
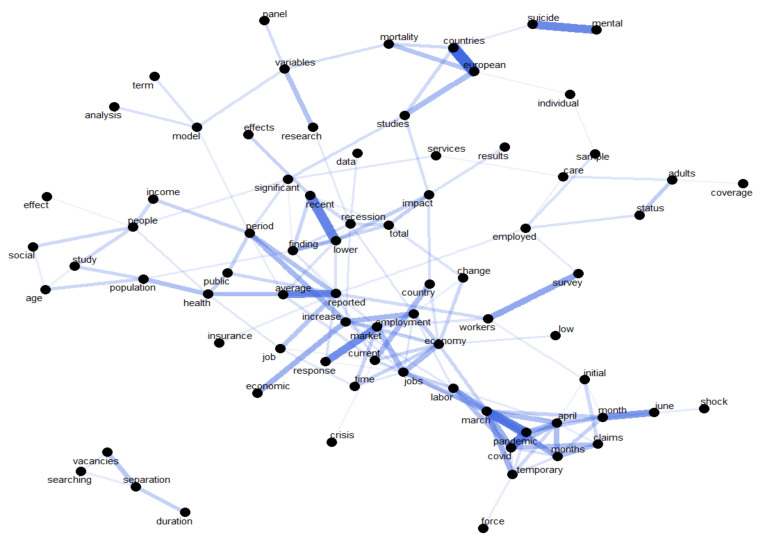
Correlation network in scientific publications’ content.

Figure 1, Figure 2 and Figure 3 offer an empirical overview of the most relevant studies in the research field of unemployment and healthcare services in the context of economic crisis using a sample of 28 research articles, covering the period 1996–2021 revealing the most common words, the relationships between words investigating which words tend to co-occur within the same documents and also the words that tend to appear more often. In exploring all these analyses, we have considered for the co-occurrence a frequency of at least 20 times while for the correlation analysis we have taken into account a correlation higher than 0.5.

Exploring the valuable information provided by the world clouds, we tried to respond to the following main research questions: What are the most common words found in the full scientific articles?

The empirical analysis proved that the most common words in the full content of selected articles apart of the keywords used “unemployment”, “economic”, “crisis”, and “health”, are the following: “COVID-19”, “data”, “labor”, “model”, “job”, “pandemic”, “increase”, “workers”, “temporary”, “population”, “recession” (Figure 1). The global COVID-19 pandemic has led to job loss of catastrophic proportions all over the world, workers facing temporary suspension or reduction in activity, therefore, COVID 19 is associated with labor market, respective unemployment. and due to the great losses. Because it represented the cause of life and economic losses, COVID-19 is associated with recession.

Analyzing which words have a co-occurrence rate of at least 20 times frequency, the empirical results highlighting the fact that unemployment rate–health–people, unemployment rate–job, unemployment rate–recession, unemployment rate–economic–period–effects, unemployment rate–economic–data-based–employment–economy, unemployment rate–increase–labor, or unemployment rate–response–labor–crisis–jobs represents the most common combinations in the most relevant studies in the field (Figure 2). Unemployment rate is associated with people, as it is characteristic to active population and health, between them existing an inverse and strong correlation, health people will register a low level of unemployment. Unemployment is related to recession; in the time of a recession unemployment registers high values. Coronavirus pandemic conduced to increase unemployment and a job crisis, lot of people temporary facing suspension or reduction in activity.

Exploring the most correlated words within the selection of articles, using as threshold the value of 0.5, it can highlight the following combinations of words being the most encountered (Figure 3): mental–suicide, European–countries–studies, workers–survey, finding–recent–lower, average–reported–period–public–health, job–reported, market–response, economic–increase–employment, market–jobs–labor–COVID–pandemic temporary–claims.

## 4. Data and Methodology

In this econometrical demarche of producing forecasts for the Romanian unemployment rate, we have used monthly data for univariate models (ARFIMA, SETAR) January 2000–April 2021, while for multivariate models, due to lack of monthly data we have used quarterly data, covering the period 2000Q1–2020Q4, the data source of the series being the dataset of European Union labor force survey, EU-LFS. For both series, we have used the same training data, even with different type of frequency, January 2000–June 2018, for estimating the parameters of the models, and the period July 2018–March 2021 to evaluate the accuracy of the models, setting out as forecast period the last two years, 2021–2022.

Therefore, in order to compare the accuracy of the results, we will aggregate monthly data into quarterly ones and we will apply Diebold–Mariano test for test data.

From a multivariate point of view, the focus of this study is also the analysis of the effect of the health crisis COVID-19 on unemployment rate in relation to healthcare services, capital stock, human capital, and economic growth. One of the characteristics of the pandemic recession is that it mainly affects the labor market and, only complementary, the stock of fixed capital.

The current COVID-19 pandemic has had a strong impact on the labor market from three main perspectives: number of jobs (through unemployment and underemployment), quality of work (through wages, or access to social protection) and through the effects on specific groups, with a higher degree of vulnerability to unfavorable labor market outcomes [8]. Therefore, decreases in incomes, increases in unemployment, financial uncertainty, reductions in living conditions, and the pressure exerted on healthcare systems are only some of the COVID-19 consequences.

From this point of view, the paper has taken into account additional variables, which have a direct or indirect relation with unemployment. Starting from the model adopted by Djurovic et al. [61] and Djurovic et al. [62], we explore the interdependencies between the following variables: unemployment, capital stock, human capital, health spending, economic growth, and economic freedom.

${\mathrm{Unemployment}}_{t}$ is the unemployment rate, ${\mathrm{Capitalstock}}_{t}$ is the gross fixed capital formation percentage of GDP, ${\mathrm{humancapital}}_{t}$ is the percentage of those employed and possessing a higher education, health spending, as percentage of GDP, denotes the current health expenditures including healthcare goods and services consumed during each year, but it does not include capital health expenditures, such as buildings, machinery, IT, and stocks of vaccines for emergency or outbreaks, ${\mathrm{economicgrowth}}_{t}$ is the percent change in quarterly real GDP and ${\mathrm{economicfreedom}}_{t}$ represents the economic freedom the overall index of economic freedom comprising ten components grouped into four broad categories: Rule of Law; Limited Government; Regulatory Efficiency; and Open Markets. The overall economic freedom is scored on a scale of 0 to 100, where 100 represents the maximum freedom. The main data sources have been the Quarterly National Accounts and Quarterly LFS of Eurostat, The Global Economy platform, as well as the Heritage Foundation.

Because COVID-19 affects symmetrically the economy, the economic growth, gross fixed capital formation, health expenditures, and economic freedom summarizes impacts on the demand side, while human capital and unemployment rate are related to effects on the supply side.

According to New Keynesian macroeconomic model, the neoclassical production function implies GDP growth, capital, and labor [63]. Okun’s Law imply the growth rate of the GDP increases employment, reducing unemployment [18]. Assuming Cobb–Douglas production technology, many labor market macroeconomic models imply unemployment is dependent on the capital stock [64]. Layard et al. [65] and Layard and Nickell [66] estimate that unemployment is affected by stock capital. Unemployment leads to negative productivity effects because the opportunities to acquire human capital through on-the-job training are lost and working habits deteriorate [67]. Musgrave’s concept of merit suggests public expenditures (education spending, welfare, and health spending) generates benefits for the society in the long term as positive externalities [68], including unemployment. Feldman [69] provided insights into the effects of economic freedom on unemployment for the first time, highlighting that economic freedom reduces unemployment. 

Although it would be considered that stock capital leads to job creation, by investing in technology, the demand for labor is reduced, increasing unemployment [70,71,72]. Increases in human capital will reduce unemployment, the relationship being inverse [73,74,75]. The relationship between health spending and unemployment is inverse, increases regarding health spending reduce unemployment [76,77,78]. The inverse relation between economic growth (GDP) and unemployment is confirmed by Kreishan [79], Calmfors and Holmlund [80], Levine [81], and Njoku and Ihugba [82]. Economic freedom reduces unemployment [83,84,85,86,87].

Therefore, the main objective of the paper is to compare the forecasting potential of univariate (ARFIMA and SETAR) vs. multivariate models (VAR/VECM) and to predict future values of unemployment rate taking into account the following steps:

1. Fitting the univariate models on the monthly training dataset covering January 2000–June 2018;
2. Fitting the multivariate models on the quarterly training dataset covering 2000Q1–2018Q2;
3. Compare the in-sample forecast accuracy measures for the all models, aggregating monthly data to quarterly data;
4. Compare the out-of-sample forecast accuracy measures for all models over the period, July 2018 to March 2021, respectively, from 2018Q3–2021Q1;
5. Compare the forecast projections of unemployment rate for all models over the period 2021Q2–2022Q4.

The data in this study are processed using software Eviews 9.5 (Quantitative Micro Software, Irvine, CA, USA) and R version 4.03 (R Foundation for Statistical Computing, Vienna, Austria).

### 4.1. ARFIMA Models

In the literature, the ARIMA (p, d, q) has been introduced by Box and Jenkins to model non-stationary times series data, who exhibit a slow decaying autocorrelation function (ACF). The order of integration d is used to model a series that is not stationary in mean, but d represents differencing with positive integer numbers. From the empirical point of view, the concept of long-term memory is usually related with the persistence showed by the autocorrelations sample of certain stationary time series, which converge to zero at a very slow pace. This behavior is not compatible with the autocorrelation functions of the models of autoregressive moving average or ARMA, which impose an exponential decrease in the autocorrelations, nor with the degree of persistence in the autoregressive integrated moving average models, ARIMA [88]. Granger and Joyeux [86] pointed out the negative consequences of differentiating the series to achieve stationarity. This is available especially in the case of over differencing, when low frequency component, which is very important in the long-term forecast, has been removed from the original series. Additionally, the alternative of not differentiating is also not appropriate because it would imply the non-stationarity of the series [89].

To solve this problem, some authors [88,90,91] introduced the so-called ARFIMA models (autoregressive fractionally integrated moving average) covering the gap between the extreme cases of unitary root models, and stationary models that impose an exponential decrease in the autocorrelations. This type of model has this particularity of using a fractional covering the “intermediate case” that exists between the unitary root ARIMA processes and the ARMA processes [92]. Therefore, ARFIMA models can be seen as a generalization of ARIMA model [89]. The ARFIMA processes produce long memory if the parameter of differentiation is in the range 0 < d < 1/2, in which case the process is stationary and invertible. In order to prove that the data follow a long-term memory pattern, the Hurst statistics can be applied [92].

In ARFIMA models, the estimation of d parameter is the key step in yielding a good model fit. The general form of ARFIMA model, based on three parameters, p is an autoregressive parameter, d is an integrated value which is a real number or not an integer, and q is a moving average parameter. ARFIMA model (p, d, q) has the following form [88,93,94]:

(1) $$\phi_{p}\left(B\right){\left(1-B\right)}^{d}Z_{t}=\theta_{q}\left(B\right)e_{t}$$

where: d = differencing parameter, $\phi_{p}\left(B\right)=\left(1-\phi_{1}B-\ldots -\phi_{p}B^{p}\right)$ is operator AR (p), $\theta_{q}\left(B\right)=\left(1-\theta_{1}B-\ldots -\theta_{q}B^{q}\right)$ is operator MA (q), ${\left(1-B\right)}^{d}$ is fractional differencing operator, $e_{t}$ is white noise process.

For this reason, the estimation methods of this parameter are divided into two main classes parametric and semiparametric methods, with different specificities.

If the parametric methods estimate all parameters of the model in one step, the semiparametric methods are carried out in two steps. First, the d parameter is estimated and secondly the parameters AR and MA are estimated. Among the most popular semiparametric method used are Geweke dan Porter-Hudak (GPH), Sperio method introduced by Reisen and Lopes [93], rescaled range statistic (R/S) or often called Hurst statistic test, as semiparametric methods, and EML is considered for the parametric approach.

For all these methods we will use R software.

According to Safitri and Ispriyanti [90], Burnecki and Sikora [91], Octaviyani et al. [92], and Maqsood and Aqil Burney [94], the main steps to be applied are the following:

a. Conducting stationarity tests in mean;
b. Identifying whether the data have long-term memory pattern using Hurst (H) statistics computed using R software. If the computed H is equal to 0.5 then the series are random, if 0 < H < 0.5 then the series shows short-term memory, and if 0.5 < H< 1 then the series shows long-term memory [95];
c. Estimating the differencing parameter (d) using parametric and semi-parametric methods. Parametric method is able to estimate all parameters in the ARFIMA model in one step [14]. In this study, the parametric method used is the exact maximum likelihood (EML) method introduced by Sowell [95]. This method uses the likelihood principal to estimate d, ϕ, and θ in the ARFIMA model.

Estimation of fractional difference parameter with semiparametric methods is carried out through two steps. The first step is estimating the fractional difference parameter (d) and the second step is estimating AR and MA parameter [14];

d. Model Diagnostic Checking (Ljung-Box and JB tests);
e. Selection of Best ARFIMA model;
f. Calculating the accuracy of the model for both training and test data.

### 4.2. SETAR Models

The SETAR model takes part from the TAR models and represents an extension of autoregressive models, bringing as its main advantage in the modeling of a time series, a higher flexibility in parameters due to a regime switching behavior. Thus, this particular type of model allows for the prediction of future values of unemployment rate, assuming that the behavior of the time series changes when the series switch the regime and this switching is dependent on the past values of the series. The model relies on an autoregressive model of lags p, on each regime and is denoted to be SETAR (k, p), where k is the number of threshold (k + 1 regime assumed in the model) and p is the order of an AR (p).

Even if the process is assumed linear in each regime, the switching from one regime to another transform the process into a non-linear one.

The general specification of a two regime SETAR (2, p, d) is the following regime to the others makes the entire proves non-linear [96,97]. The two-regime version of the SETAR model of order p is given by:

(2) $$y_{t}=\phi_{0}^{\left(1\right)}+\sum_{i=1}^{p\left(1\right)}\phi_{i}^{\left(1\right)}y_{t-i}+{Ɛ}_{t}^{\left(1\right)},\;\mathrm{if}\;y_{t}-{\;}_{d}\;\leq \;\tau$$

(3) $$y_{t}=\phi_{0}^{\left(2\right)}+\sum_{i=1}^{p\left(2\right)}\phi_{i}^{\left(2\right)}y_{t-i}+{Ɛ}_{t}^{\left(2\right)},\;\mathrm{if}\;y_{t}-{\;}_{d}\;>\;\tau$$

where $\phi_{i}^{\left(1\right)}$ and $\phi_{i}^{\left(2\right)}$ are the coefficient in lower and higher regime, respectively, which needs to be estimated; τ is the threshold value; $p\left(1\right)$ and $p\left(2\right)$ are the order of the linear AR model in low and high regime, respectively. y_t__− d_ is the threshold variable governing the transition between the two regimes, d being the delay parameter which is a positive integer (d < p); ${Ɛ}_{t}^{\left(1\right)}$ and ${Ɛ}_{t}^{\left(2\right)}$ are sequence of independently and identically distributed random variable with zero mean and constant variance [98].

The main phases for setting a SETAR model are order selection of the model based on AR (p) order identification together with the test for threshold non-linearity, model identification requiring the selection of the delay parameter d together with the location the threshold value, model estimation, and evaluation and the last stage forecasting the future values of unemployment rate. 

Thus, the first stage in applying the SETAR model, is to analyze the existence of a non-linearity behavior and for that it is important to first determine the appropriate lag length of the autoregressive model AR (p) for the analyzed time series and the choice could rely on the minimum value of AIC. Secondly, we will test the existence of non-linearity using the Tsay F test, the null hypothesis of linearity being rejected if the *p*-value of the test is smaller than the significance level assumed.

Proving that there is non-linearity in the time series, we can pass to the second stage—model identification—and we will consider a two regime SETAR model with the order p of autoregressive parts equal in both regimes, SETAR (2, p, d).

In the third stage, the selection of delay parameter together with the location of the threshold value is realized, taking into account that the possible value d is less than order. Therefore, several SETAR models, with different delay parameter and threshold value can be identified and based on a grid search method, the best model is selected to be the model with the smallest value for residual sum of squares.

The model is estimated using the MLE and then the adequacy of the selected model is evaluated based on diagnostics tests on residuals. The ARCH-LM test is used for testing the hypothesis of constant variance and Breusch–Godfrey is used for testing for higher-order serial correlation in the residuals.

### 4.3. VAR/VECM Models

In order to predict multiple time series variables using a single model, a vector autoregressive (VAR) is used, representing an extension of the univariate autoregressive model to dynamic multivariate time series [99].

Instead of using only its own past to predict the future, we adopted also multivariate approach adding also variables with a direct or indirect relation with unemployment starting from the model of Djurovic et al. [61] and Djurovic et al. [62] and exploring the dynamic interdependencies between unemployment, capitol stock, human capital, health spending, economic growth, and economic freedom, as we previously discussed. Therefore, we have used the non-stationarity tests, Johansen cointegration test, and VAR/VECM models.

The non-stationarity analysis implied the usage of both ADF and PP unit roots tests, in order to reveal the order of integration of the variables. If all the variables are integrated of the same order, it makes sense to go further and to investigate a potential long-run relationship among them using a VAR framework in level for which the optimal lag need to be identified based on AIC or SCH assuming the absence of a serial correlation in the residuals. If the variables are not cointegrated, a VAR model in difference must be estimated; otherwise, if the variables are integrated of the same order, an error correction mechanism need to be incorporated within a VAR model.

The general form of a VAR model is the following:

(4) $$X_{t}=A_{1}X_{t-1}+\ldots +A_{p}X_{t-p}+\mu +\varepsilon_{t}$$

where: X_t_, X_t − 1_, …, X_t − p_ are (n × 1) vectors (x_1t_, x_2t_, …, x_nt_)′ of current and lagged values of n variables which are I(1) in the model; A_1_, …, A_p_ are matrices of coefficients with (n × n) dimensions; μ is an intercept vector; $\varepsilon_{t}$ is an independently and identically distributed vector with zero mean and variance matrix Σ_ε_ [100].

The ECM restricts the long-run behavior of the endogenous variables to converge to their cointegrating relationships while allowing for short-run adjustment dynamics. The cointegration term is known as the correction term since the deviation from long-run equilibrium is corrected gradually through a series of partial short-run adjustments [101].

The ECM estimated can be defined as follows:

(5) $$\Delta X_{t}=\mu +\Gamma_{1}\cdot \Delta X_{t-1}+\ldots +\Gamma_{p-1}\Delta X_{t-p+1}+\Pi \cdot X_{t-1}+\varepsilon_{t}$$

where: Δ is the difference operator, X is a vector formed by the n variables used in our currency demand. Γ denotes an (n × n) matrix of coefficients and contains information regarding the short-run relationships among the variables. Π is an (n × n) coefficient matrix decomposed as Π = γ·β′, where γ represents the adjustment coefficients and β the cointegrating vectors.

It is important to assess the adequacy of the model by performing diagnostic checks to determine whether the residuals of the model approximate white noise.

### 4.4. Forecasting Performance Comparison

In order to choose the best model of providing forecasts of unemployment rate, all three models have been compared for the out of sample period, evaluating the forecasting accuracy: the root mean squared error (RMSE), the mean absolute error (MAE), and the mean absolute percent error (MAPE). The better forecast performance of the model is that with the smaller error statistics. 

Additionally, the Diebold–Mariano test [102] has been applied to evaluate the statistically significant differences between the forecast accuracy of every two models. In order to do that, we have aggregated our data, transforming the monthly forecasts into quarterly forecasts, averaging the values of time series.

## 5. Empirical Results

After 2000 unemployment rate presented an oscillate trend, with values from 5% (September 2008) to 8.1% (January 2001 and January–March 2002)). In the winter (2000, 2001 and 2002) unemployment increased as a result of lack of jobs, reaching values of 144% (2002). This was the response of implementation of restructuring and privatization programs in different sectors of activity. 

In 2004, the unemployment rate decreased until 2006 due to legal and illegal migration in order to work abroad, in 2006 Eurostat estimated over two million Romanians working in other countries. In 2008, unemployment decreased, but the economic crisis contributed to increasing unemployment, in 2010 reaching the values bigger than of 7.7%, this trend was maintained until 2010.

Unemployment rate decreased in 2019, affecting the graduates of lower and secondary education most, the unemployment rate for people with higher education being lower.

In the most recent period, unemployment rate continuously decreased, in January 2020 being 3.8%, but starting February 2020 with an increased unemployment rate due to high unemployment among young people and seasonal factors (construction and tourism).

Since February 2020 in the context of the coronavirus crisis, unemployment increased, in May being registered the highest level since 2017. In August, for the first time since COVID-19 affected the Romanian economy, unemployment decreased, in October it started to increase again.

**Figure 4:**
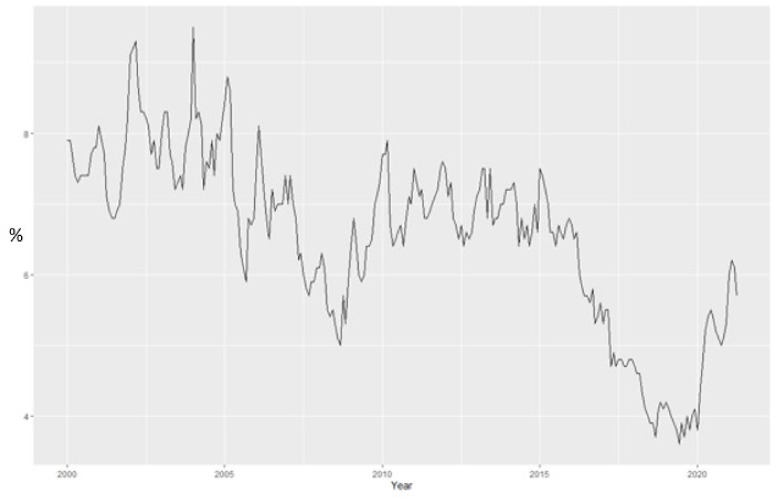
The Romanian ILO unemployment rate for the period 2000M1–2021M04.

In 2021, unemployment increased in January and February, reaching values of 5.6% and 5.7%, decreasing in March to 5.5% and 4.4% in May (Figure 4).

**Figure 5:**
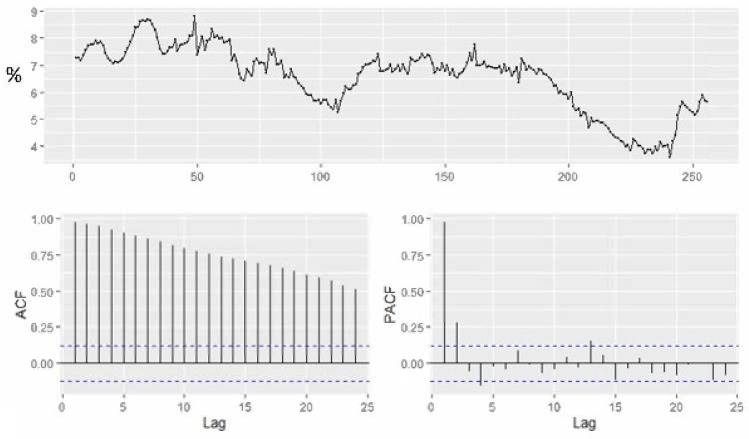
Autocorrelation and partial correlation plot of Romania’s monthly unemployment rate for the period 2000–2021.

**Figure 6:**
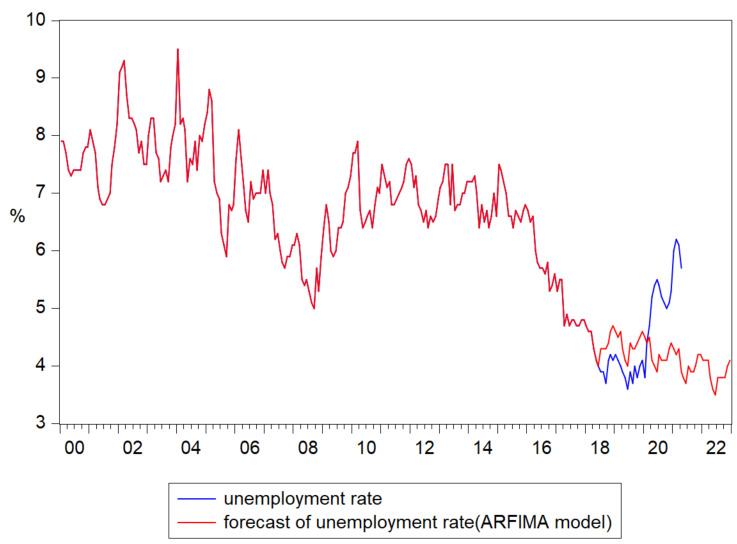
Forecasts of unemployment rate based on the results of ARFIMA ([10], 0.245, 0) model.

Figure 5 revealed that the Romanian unemployment rate exhibits a weak seasonal fluctuation over the period 2000–2020 and the series has been seasonally adjusted with Census X-11 method. Figure 6 presents the monthly evolution.

### 5.1. ARFIMA Model

After removing seasonality, we observe the ACF decays at a hyperbolic rate, exhibiting a significant dependence between the observations. It reveals the indication of presence of long memory in the series. For fitting an ARFIM model, we used monthly data covering the period January 2000 to June 2018. Augmented Dickey–Fuller (ADF) and Phillips–Peron tests (Table 1) have been conducted to find out if the data are stationary in mean, the empirical results revealing that the data are not stationary in mean.

In order to identify the long-term pattern and to detect if the series has long-memory, the ACF plot together with the Hurst statistics value can be evaluated. According to the autocorrelation plot, the time series has a long memory since the coefficients decreased slowly and hyperbolically. According to Hurst statistics through R/S statistics (The computation of Hurst exponent has been performed in R, using the function hurstexp), the value lies between 0.5 and 1, leading to the conclusion that there is long memory pattern in the data (Table 2).

The ARFIMA model is, therefore, used to capture long-range dependence among the observations rather than applying the procedures used for short-term memory. The first step in constructing the ARFIMA model is to obtain the estimate of fractionally differenced parameter ‘d’ which is a long memory parameter. For this purpose, we have applied both parametric and semiparametric methods.

In building the ARFIMA model with a parametric approach, the candidate models can be identified from the plot of ACF and PACF of the differenced series. A temporary d value is obtained by fitting ARFIMA (0, d, 0) model. The estimated d is 0.494.

To identify the order of p and q as ARFIMA model, the value of d is set to 0.494. According to the plot of ACF and PACF, the model candidates are ARFIMA (2, d, 0), ARFIMA (3, d, 0), ARFIMA ([9], d, 0), ARFIMA ([3,10], d, 0), ARFIMA (0, d, 2), ARFIMA (0, d, 3), ARFIMA (1, d, 1), and ARFIMA (2, d, 2). The parameters for each candidate model are then estimated simultaneously by using EML method. Table 3 summarizes the estimated parameters for each model. The selection of the optimal model has been based on the statistical significance of the parameters and on AIC. It shows that ARFIMA ([3,10], 0.321, 0) has the lowest AIC value (Table 3).

In semiparametric method, the fractional difference parameter is estimated separately from the AR and MA coefficients (Table 4). Table 5 presents the estimated value of d by using the three semiparametric methods using R software. All estimators returned similar positive values, which indicates the long memory property.

Next, we fractionally differentiate the series considering the memory parameter d = 0.245, which is the average value of obtained semi-parametric estimators [96].

Next. we established an ARMA model to fractionally differenced series, we chose the strategy developed by Box and Jenkins. Model identification as ARFIMA (p, d, q) was conducted by using the plot of ACF dan PACF. We took different combinations of autoregressive polynomial p and moving average polynomial and select the best pair on the basis of some criterion measures, such as Akaike information criterion (AIC) and Schwarz criterion (SC). The candidate models are ARFIMA (2, d, 0), ARFIMA (3, d, 0), ARFIMA (4, d, 0), ARFIMA (9, d, 1), ARFIMA ([10], d, 0), ARFIMA (1, d, 1), ARFI-MA (2, d, 1), and ARFIMA (2, d, 2). Table 5 summarizes the comparison of all models with their corresponding AIC values. The smallest AIC values are for ARFIMA ([10], d, 0).

Apart of classical tests, *t*-test for the statistical significance of the parameters and F-test for the validity of the model, the selection of the best model depends also on the performance of residuals. For that, the series of residuals has been investigated to follow a white noise. For checking the autocorrelation of residuals, we have applied serial correlation LM test, while autoregressive conditional heteroskedasticity, the ARCH-LM test has been used, the empirical results confirmed that there is no autoregressive conditional heteroscedasticity (ARCH) in the residuals. Diagnostic model checking revealed that the candidate model based parametric and semiparametric methods show a good fit model since none of the assumptions are violated. 

Next, we examined both models in terms of accuracy by using MAE, MAPE, RMSE, and MASE. Table 6 shows that the smallest values, especially for the testing dataset, are for ARFIMA ([10], d, 0) with d = 0.245.

ARFIMA ([10], d, 0) model with d = 0.245 can be expressed as follows:

$$\left(1+0.173\cdot B^{10}\right){\left(1-B\right)}^{0.245}Y_{t}=e_{t}$$

The forecast of the unemployment rate based on the ARFIMA ([10], 0.245, 0) model results highlighted an downward trend for the last period, reaching the value of 4.5% in March 2020 and decreased to almost 4.1% in December 2022 (Figure 6).

### 5.2. Self-Exciting Threshold Autoregressive (SETAR Model)

**Figure 7:**
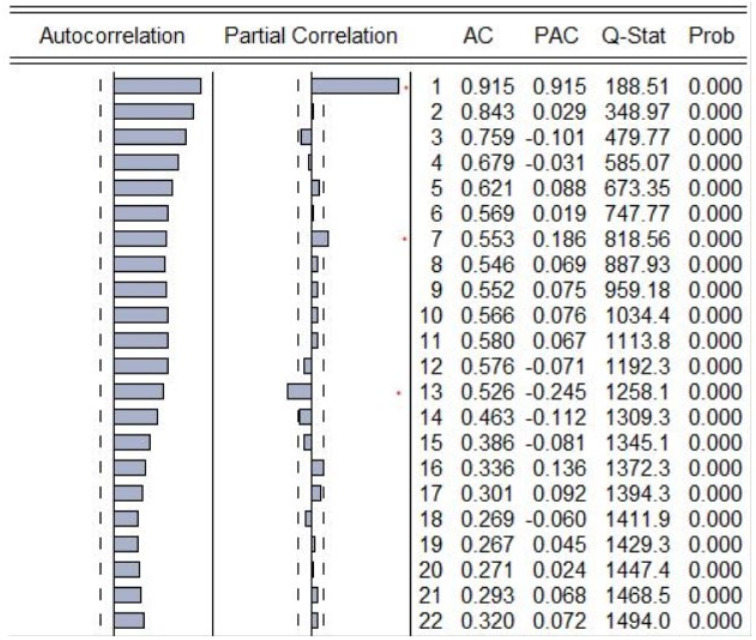
Partial autocorrelation plot of unemployment series.

Verifying the existence of non-linear thresholds represents an important stage in setting up a SETAR model. In order to do that, the autoregressive order has been identified based on both PACF plot and Tsay test. It is worthwhile to mention that the non-stationary property of the unemployment series does not occur for the non-stationary of non-linear thresholds in autoregressive of SETAR model [71]. Analyzing the partial correlation plot, significant spikes can be observed at lag 1, 7, and 13. (Figure 7)

Moreover, at these lags, the existence of nonlinear thresholds has been tested with the Tsay test, the empirical results highlighting non-linear threshold at lags 1, 7, 8, 9, 10, 11, 12, 13, with very small *p*-values, less than 1%. Therefore, we can assume that at these particular lags, the SETAR model provides better results than the simple autoregressive model (Table 7).

From all these lags exhibiting non-linear thresholds, based on the lowest value of AIC, the optimal model AR(13) has been selected for developing a SETAR models, considering the potential values of delay parameter d = 1…12 < p.

**Figure 8:**
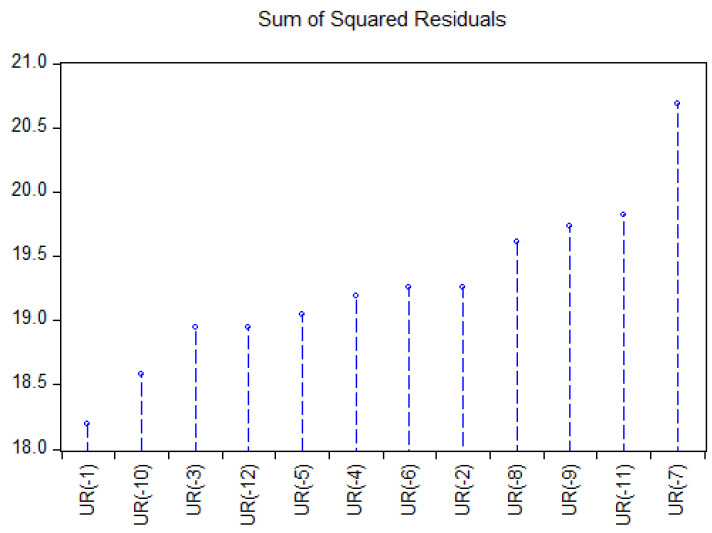
Grid search method estimation of one threshold value.

Assuming the correspondence between the number of regimes and the number of threshold values, a grid search method has been performed to identify the number of the regimes and to estimate thresholds value, acknowledging the criterion of the minimum value of sum squared residuals (Figure 8).

Therefore, the optimal decay parameter can be considered d = 1, with the smallest value of SSR and a SETAR model (2, 13, 1), two regimes of order 13 and threshold decay 1 have been used to explore the non-linearity within the unemployment rate (Table 8).

The estimated model of SETAR (2, 13, 1) with the threshold of 7.79 is presented in Table 6, the model having the following specification:

$$y_{t}=\left\{\begin{array}{c} 0.005+0.83y_{t-1}+\ldots -0.297y_{t-13},\;if\;y_{t-1}<7.799\\ 2.344+0.539y_{t-1}+\ldots -0.019y_{t-13},\;if\;y_{t-1}>7.799 \end{array}\right\}$$

The stage of residuals diagnostics revealed that the errors are white noise, the Breusch–Godgrey test revealing at lag 12 the absence of serial correlation, while the ARCH-LM test proved the absence of autoregressive conditional heteroscedasticity, in both cases the probabilities being much greater than the threshold of 10% (Table 9).

**Figure 9:**
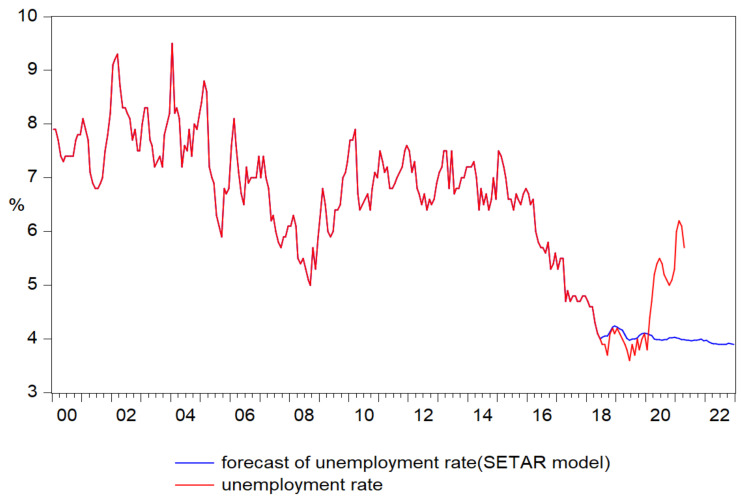
Forecasts of unemployment rate based on the results of SETAR (2, 13, 1) model.

The forecast of unemployment rate based on the results of SETAR (2, 13, 1) model faithfully reproduced the pattern of the original series registering in February 2020, just before the outbreak of the pandemics the value of 4.1%, from this point the forecast follows a totally divergent trend in comparison with the original series. Thus, if the original series of the unemployment rate registered during 2020, a predominantly upward trend that reaches its maximum in February 2021 (6.2%), the forecast oscillates around 4% with slight trends of increase and decrease, reaching the value of 3.9% at the end of 2022 (Figure 9).

### 5.3. VAR/VECM Model

From this point of view, the paper has taken into account additional variables, which have a direct or indirect relation with unemployment. Starting from the model adopted by Djurovic et al. [61] and Djurovic et al. [62], we estimated the following model:

(6) $$\begin{array}{ll} {\mathrm{Unemployment}}_{t}= & \beta_{0}+\beta_{1}\cdot {\mathrm{Capitalstock}}_{t}+\beta_{2}\cdot {\mathrm{humancapital}}_{t}+\beta_{3}\cdot {\mathrm{healthspending}}_{t}+\beta_{4}\cdot {\mathrm{economicgrowth}}_{t}+\beta_{5}\\ & \cdot {\mathrm{economicfreedom}}_{t} \end{array}$$

The first step in building a VAR/VECM model, is the non-stationarity analysis realized based on ADF and PP unit root tests for all three scenarios (trend and intercept, intercept and drift) revealing that all variables are I(1), being non-stationary. The series gross fixed capital formation initially exhibited a seasonal pattern and the series has been seasonally adjusted using Census X-13.

Having all variables integrated of the same order, it makes sense to test the presence of a long-run equilibrium relationship using the multivariate Johansen cointegration test under a VAR model at level. The optimal lag has been identified based on the informational criteria SC and HQ, verifying simultaneously the stability condition according to which absolute values of all eigenvalues of the system matrix lie inside the unit circle and also the hypotheses on residuals (non-autocorrelation, homoscedasticity and normality), the empirical results pointing out as optimal lag, lag 1.

Based on this framework, the empirical results of Johansen approach revealed the presence of a unique long-run relationship between unemployment rate, capital stock, human capital, health spending, economic growth and economic freedom, at 1% significance level.

Thus, in the light of these results, the VAR model should include a mechanism of error correction model (ECM) and the empirical results of the VECM model are presented in the Table 10.

The empirical evidence highlighted the statistical significance of coefficients for the long-run relationship, a 1% increase in economic growth would imply an estimated decrease of almost 0.88% in unemployment rate, ceteris paribus. Additionally, a 1% increase in health spending would contribute to a decrease of about 1.434% in unemployment rate, ceteris paribus, while a 1% increase in economic freedom would contribute to a decrease of about 0.927% in unemployment rate, ceteris paribus. Therefore, it is important to mention that in long run economic growth, health spending and economic freedom exhibited a negative impact on the unemployment rate, while the capital stock manifested a positive impact, revealing that 1% increase in capital stock would conduct to an increase of about 0.383%, ceteris paribus. The human capital does not provide any impact on the unemployment rate.

The error correction term, capturing the speed of adjustment to disequilibrium (error correction term, ECT) highlighted that the deviation from the long-term equilibrium of unemployment rate is corrected by 10.2% over each quarter.

The negative sign and the statistical significance of the coefficient ECT offers valuable information about the existence of a long-run causality running from all variables included in the analysis to unemployment rate.

However, in the short-term, the empirical results invalidated almost all the potential relationship between variables, due to the lack of statistical significance of the variables, with the exception of unemployment rate from the previous quarter who exhibited a positively and statistically significant impact on the current unemployment rate.

The results of VECMs indicate that economic freedom, economic growth, health spending, and capital stock explain about 43% of the variation on unemployment rate, F-statistic supporting the model is correct specified. The percentage of the total variation of the dependent variable is considered high enough (44.89%) for the model. 

As expected, the coefficients economic growth, health spending and economic freedom present a negative long-term economic effect on the unemployment rate. Therefore, according to Levine [81], Kreishan [79], and Li and Liu [103] economic growth causes obvious to decrease unemployment rate. Pirim [76], Mattei and Pistoresi [104], and Suyanto et al. [105], indicated that an increase in health spending will cause a decrease in unemployment rate and Feldman [69], Pearson et al. [106], and Cebula et al. [107] demonstrated that economic freedom through economic growth is conducive to a decrease in unemployment rate, as well.

Regarding capital stock, Arestis and Biefang-Frisancho [67], Karanassou and Snower [108], and Rowthorn [70] highlighted that the result of increasing the capital stock will be an increase in unemployment rate due to automation and digitization.

The diagnostics of residuals revealed that the VECM errors are non-autocorrelated and homoscedastic, based on the results of Breusch–Godfrey test and White test, in both cases the probabilities being much greater than the threshold of 10% (Table 11).

**Figure 10:**
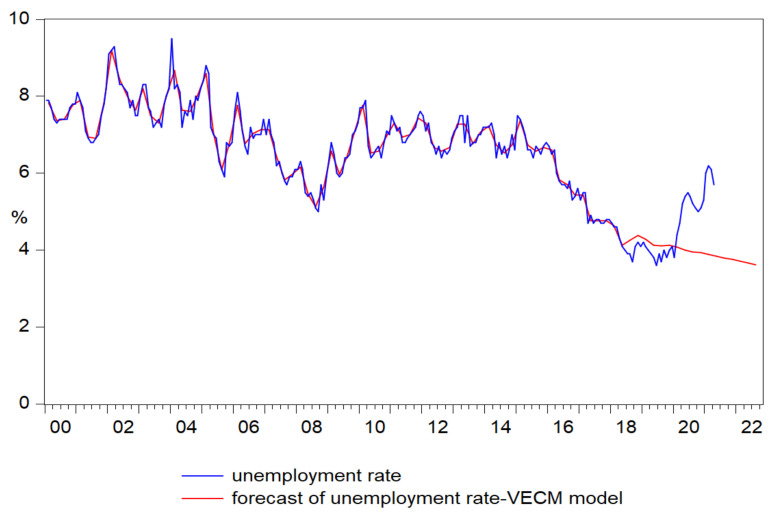
Forecasts of unemployment rate based on the results of VECM model.

**Figure 11:**
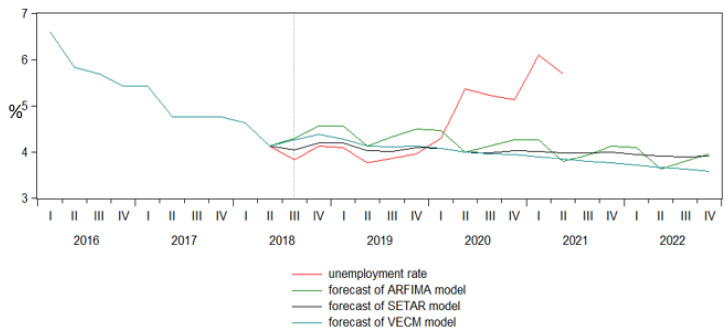
Forecast comparison graph of unemployment rate.

The forecast of unemployment rate based on the results of VECM model (Figure 10) faithfully reproduced the pattern of the original series until 2020Q1, registering the value of 4.1% and following a slowly decreasing trend reaching the value of 3.6% at the end of 2022 (Figure 11).

### 5.4. Comparison of Models Forecasting Performance

Analyzing the forecasting performance of all three models for an out of sample dataset based on RMSE, MAE, and MAPE, as well as on the results of the Diebold and Marino test, it is important to mention that after aggregating the monthly data to quarterly data, the model who provides the most reliable results for the test data set is SETAR, with the smallest values for the evaluation statistics, as individual method (Table 12). 

The empirical results of Diebold–Mariano test confirmed the existence of a statistical difference in the accuracy of ARFIMA and SETAR models, for the out of sample data, rejecting the null hypothesis having the probabilities very closed to 0. Furthermore, VECM and SETAR models seems to have similar forecast accuracy, with a very high probability of the test (Table 13).

Therefore, we can conclude that SETAR and VECM models offer very closed forecasts for the period 2018–2022, but in terms of accuracy the best solution is the SETAR model provided better results, leading to a projection of unemployment rate to almost 3.9%.

**Figure 12:**
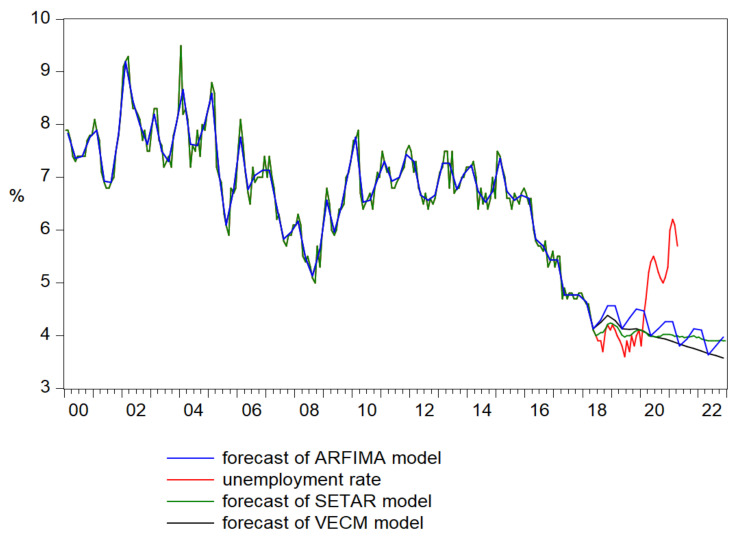
The forecasts of unemployment rate for the period 2021–2022.

Therefore, analyzing the accuracy of univariate vs. multivariate models in forecasting the unemployment rate, it is very clear that the SETAR model as a univariate model provided the most reliable results, both for replicating the pre-pandemic period, 2018Q2–2020Q1, reaching the value of 4.1% at the beginning of 2020, so that later the trend to become disjointed (Figure 12). 

Therefore, if the pandemics produced a raise in the unemployment rate to almost 6.1% in 2021Q1, the SETAR follows a decreasing trend, reaching the value of 3.9% at the end of 2022. In conclusion, it is worthwhile to mention that our empirical results fully supported the main research hypothesis.

## 6. Discussions

The health crisis caused by COVID-19 has generated a sudden and deep recession around the world, testing health and social protection systems, societies and economies, the way we live and collaborate. The effects and prospects for recovery are uneven from country to country depending on the spread of the virus, the strictness of public health measures taken in order to keep it under control, the sectoral composition of national economies and the strength of national policy responses.

One of the measures taken was the balance between the need for fiscal adjustment and the need to support economic recovery, supporting the economy and the health system, remaining a priority in the current difficult circumstances.

In the context of the pandemic, states, including Romania, have implemented important programs to support the economy and maintain the standard of living. Fiscal measures have been adopted in order to cover the financial needs in the sectors most affected by the COVID-19 pandemic crisis, in particular to support employees or ensure a minimum income for the unemployed, as well as to avoid bankruptcy, a very large number of firms, especially SMEs, are essential to avoid a more severe contraction of economic activity.

In order to protect the persons who have lost their jobs and support those who work, but also to reduce the pressure on national public finances, thus strengthening the social dimension of Europe, the European Commission has proposed the implementation of European Unemployment Reinsurance Scheme. Thus, national policies for jobs and skills protection are supported through work schemes with reduced programs or facilitating the transition of the unemployed from one job to another.

The crisis caused by the COVID-19 pandemic and the impact suffered by the sudden and almost general deterioration of the macroeconomic context and the business environment, severely affected the extremely fragile balance of the Romanian labor market, under the action of divergent influences, at the confluence between the determinants of labor demand and supply.

According to the UN report, the loss of jobs due to the pandemic has cancelled “five years of progress in combating global poverty”, noting that, until at least 2023, “employment growth will be insufficient to compensate for the losses suffered”. The massive loss of jobs has exacerbated global inequality, with women, young people and informal sector workers being the hardest hit. The world of work will be different. On the current trajectory, as the labor market begins to recover, there is a major risk of accentuating inequality. 

Destruction of global value-added chains, total or partial closure of international trade corridors and borders, temporary bans on economic activities in most sectors of activity, hygiene and social distancing rules, restriction of the right to leave the home, and bans on leaving localities can be mentioned as some of the factors with significant influence.

These factors have affected virtually all members of society, not just those active with legal forms on the labor market or who carried out their activity in a “grey” area of it, but also important dependent population segments of them.

In addition to the general characteristics of occupations in market conditions, the Romanian labor market, also has particular characteristics, of which, at least two are important for setting up anti-crisis measures in the context of the COVID-19 pandemic, they are: the large share of unpaid activities of family workers; and the high share of wage labor without employment contracts written and recorded, informal wage labor that is unregistered and untaxed. These categories of employed people, together with self-employed workers, represent the most vulnerable categories in the face of the crisis, but also the categories often overlooked in public strategies and policies for mitigating the effects of the health crisis [109].

Additionally, ILO draw attention to the disproportionate impact of COVID-19, the most vulnerable being vulnerable groups and the informal area of the economy, those who work in the most affected sectors and low-skilled workers [110].

Therefore, as Palacios and Robalino [111] already pointed out by analyzing ways to reduce distortions of the labor market, the integration of social insurance systems with those of social assistance becomes more important having in view of the future characteristics of the labor market, in which we will have a persistence of low qualification level jobs.

## 7. Conclusions

In the context of the deterioration of the macroeconomic and financial framework, the proportions of which are difficult to predict in terms of size, depth, and extent over time, despite anti-crisis measures and government support to the economy, in the short term the labor market faced increases in unemployment, at least in 2020, as a result of the restriction of activity in many branches. On the other hand, in a favorable relaunch of the Romanian economy, allowing a rapid recovery related to the levels of the pre-crisis period, in 2021, employment problems and labor market tensions reappeared, albeit to a lesser extent.

In this context, the paper aimed to offer valuable forecasts for the Romanian unemployment rate using univariate vs. multivariate time series models for the period 2021–2022, highlighting the main patterns of evolution based on which relevant strategies for the Romanian labor market can be drawn

The empirical results pointed out that both SETAR and VECM provide very similar results in terms of accuracy replicating very well the pre-pandemic period, 2018–2020, with a decreasing trend at the end of 2022. Therefore, if the pandemics produced a raise in the unemployment rate in the first quarter of 2021, SETAR and VECM models followed a decreasing trend until the end of 2022.

In conclusion, our research supported the idea that the unemployment rate in the near future is expected to decrease after pandemic shock damping,

As already mentioned, starting this crisis, the labor market will be different. On the current trajectory, as the labor market begins to recover, there is a major risk of accentuating inequality. In this context the research it proves even more its utility contributing to the most recent forecast of one of the core indicators of the Romanian labor market, widely used in specific labor market strategies.

## Figures and Tables

**Table 1:** Unit root analysis of the Romanian unemployment rate.

Variable		Level	
		ADF	PP
Unemployment rate	T&C	−3.24 *	−3.18 *
	C	−2.08	−2.19
	None	−1.01	−1.11

Note: ***, **, * means stationary at 1%, 5% and 10%; T&C represents the most general model with a constant and trend; C is the model with a constant and without trend; None is the most restricted model without a drift and trend. For the ADF test, the number of lags was determined using SCH criterion for maximum 14 lags to remove serial correlation in the residuals. For both PP test, the value of test was computed using Newey–West Bandwidth (as determined by Bartlett–Kernel). Tests for unit roots have been carried out in E-VIEWS 11.

**Table 2:** Long memory identification and Hurst value for training data.

Simple R/S Hurst estimation	0.684
Corrected R over S Hurst exponent	0.762
Empirical Hurst exponent	0.790
Corrected empirical Hurst exponent	0.750
Theoretical Hurst exponent	0.557

**Table 3:** Estimated parameter of ARFIMA (p, d, q) using EML method.

No	ARFIMA Model (p, d, q)	Parameter	Coefficient	SIG.	AIC
1.	(2, d, 0)	Φ1	0.487	0.000	−380.86
		Φ2	0.328	0.000	
		d	0.127	0.243	
2.	(3, d, 0)	Φ1	0.225	0.098	−382.16
		Φ2	0.289	0.000	
		Φ3	0.182	0.026	
		d	0.362	0.003	
3.	([9], d, 0)	Φ9	−0.176	0.010	−389.60
		d	0.372	0.705	
4.	([3,10], d, 0)	Φ3	0.266	0.00	−393.12
		Φ10	−0.197	0.008	
		d	0.321	0.007	
5.	(0, d, 2)	Ө1	−0.072	0.389	−360.23
		Ө2	−0.277	0.000	
		d	0.485	0.000	
6.	(0, d, 3)	Ө1	−0.075	0.323	−382.61
		Ө2	−0.265	0.000	
		Ө3	−0.367	0.000	
		d	0.470	0.000	
7.	(1, d, 1)	Φ1	0.881	0.000	−376.82
		Ө1	0.323	0.000	
		d	0.116	0.410	
8.	(2, d, 2)	Φ1			
		Φ2			
		Ө1			
		Ө2			
		d			

**Table 4:** Estimated value of d using semiparametric method.

Method	Parameter d
Geweke dan Porter-Hudak (GPH)	0.238
Smoothed GPH (Sperio)	0.263
R/S	0.236

**Table 5:** Comparison of ARFIMA model using semiparametric method.

No	Semi-Parametric Method	Arfima Model	AIC
1	d = 0.245	ARFIMA (2, d, 0)	212.93
2		ARFIMA (3, d, 0)	211.29
3		ARFIMA (4,d, 0)	207.80
4		ARFIMA (9, d, 0)	204.81
5		ARFIMA ([10], d, 0)	200.33
6		ARFIMA (1, d, 1)	217.13
7		ARFIMA (2,d, 1)	213.18
8		ARFIMA (2, d, 2)	205.10

**Table 6:** Forecasting accuracy.

	dEML = 0.494		dSEMI-PAR = 0.245	
	Training Data Set	Testing Data Set	Training Data Set	Testing Data Set
RMSE	6.834	5.057	0.366	4.688
MAE	6.775	5.08	0.278	4.620
MAPE	52.43	187.42	163.939	177.41
MASE	24.21	26.28	0.799	15.26

**Table 7:** The empirical results of Tsay test.

Order	F-Statistics	*p*-Value	AIC
AR(1)	4.798	0.029 **	0.734
AR(2)	1.935	0.125	-
AR(3)	1.363	0.231	-
AR(4)	1.097	0.366	-
AR(5)	1.119	0.341	-
AR(6)	1.267	0.202	-
AR(7)	1.744	0.016 **	0.689
AR(8)	1.994	0.001 ***	0.693
AR(9)	2.116	0.001 ***	0.697
AR(10)	1.989	0.001 ***	0.696
AR(11)	2.151	0.001 ***	0.698
AR(12)	2.257	0.001 ***	0.702
AR(13)	2.034	0.003 ***	0.628

Note: ***, **, * means statistical significance at 1%, 5%, 10%.

**Table 8:** Estimates of parameters for SETAR (2, 13, 1).

Variable	Coefficient	Std. Error	t-Statistic	Prob.
UR(−1) < 7.7999999--177 obs				
C	0.005447	0.227242	0.023968	0.9809
UR(−1)	0.832909	0.076657	10.86543	0.0000 ***
UR(−2)	0.249011	0.097484	2.554390	0.0115 ***
UR(−3)	−0.056570	0.097475	−0.580355	0.5624
UR(−4)	−0.132460	0.097057	−1.364771	0.1740
UR(−5)	0.061211	0.091125	0.671723	0.5026
UR(−6)	−0.129216	0.090665	−1.425209	0.1558
UR(−7)	0.080539	0.091115	0.883926	0.3779
UR(−8)	0.074441	0.095547	0.779103	0.4369
UR(−9)	0.008233	0.091085	0.090392	0.9281
UR(−10)	−0.021469	0.095542	−0.224708	0.8225
UR(−11)	0.093245	0.098039	0.951105	0.3428
UR(−12)	0.235726	0.095365	2.471826	0.0144 ***
UR(−13)	−0.297722	0.074153	−4.014963	0.0001 ***
7.7999999 ≤ UR(−1)--32 obs				
C	2.344367	2.065582	1.134967	0.2579
UR(−1)	0.538905	0.199478	2.701573	0.0076 ***
UR(−2)	0.213822	0.181608	1.177385	0.2406
UR(−3)	0.022186	0.191967	0.115571	0.9081
UR(−4)	−0.642696	0.204075	−3.149317	0.0019 ***
UR(−5)	0.681679	0.330818	2.060589	0.0408 **
UR(−6)	0.062262	0.260761	0.238771	0.8116
UR(−7)	0.141311	0.294796	0.479353	0.6323
UR(−8)	−0.638746	0.263496	−2.424123	0.0163 **
UR(−9)	−0.526172	0.297643	−1.767796	0.0788 *
UR(−10)	0.478538	0.215476	2.220838	0.0276 **
UR(−11)	0.833487	0.178725	4.663510	0.0000 ***
UR(−12)	−0.439931	0.253990	−1.732077	0.0850 *
UR(−13)	−0.019223	0.195508	−0.098322	0.9218
R-squared	0.916210	Mean dependent var		6.787081
Adjusted R-squared	0.903711	S.D. dependent var		1.021629
S.E. of regression	0.317017	Akaike info criterion		0.664382
Sum squared resid	18.19044	Schwarz criterion		1.112158
Log likelihood	−41.42788	Hannan-Quinn criter.		0.845420
F-statistic	73.30213	Durbin-Watson stat		2.087803
Prob(F-statistic)	0.000000			

Note: ***, **, * means statistical significance at 1%, 5%, 10%.

**Table 9:** Forecasting performance of SETAR (2, 13,1).

	Training Data Set	Testing Data Set
RMSE	1.423	0.963
MAE	1.221	0.679
MAPE	17.445	13.057
MASE	-	-

**Table 10:** The empirical results of VECM model.

Long-Run Results	
Unemployment rate(−1)	1.00
Economic growth(−1)	−0.88 ***
	(0.241)
	[−3.648]
Health spending(−1)	−1.434 *
	(0.718)
	[2.00]
Economic freedom(−1)	−0.927 ***
	(0.140)
	[−6.620]
Human capital(−1)	−0.048
	(0.156)
	[−0.308]
Capital Stock(−1)	0.383 ***
	(0.05)
	[6.979]
@trend(00Q1)	−0.261 ***
	(0.042)
	[−6.085]
C	−55.369
**Short-Run Results**	
ECTt-1	−0.102 ***
	(0.025)
	[−3.96]
D(unemployment rate(−1))	0.307956 ***
	(0.13363)
	[2.30457]
D(economic growth(−1))	0.085102
	(0.03640)
	[2.33791]
D(heath spending(−1))	−0.438867
	(0.28085)
	[−1.56265]
D(economic freedom(−1))	−0.035104
	(0.05934)
	[−0.59158]
D(human capital(−1))	−0.080400
	(0.04991)
	[−1.61074]
D(capital stock(−1))	0.023179
	(0.01292)
	[1.79427]
C	−0.011339
	(0.05595)
	[−0.20268]
R-squared	0.448900
Adj. R-squared	0.388624
F-statistic	7.44 ***
The short-run diagnostic test statistics (At lag 4.)	
Autocorrelation LM test	45.34 [0.136]
Normality test	725.77 *** [0.00]
White test	731.90 [0.52]

Note: Standard errors are in parentheses. [] denote the *t*-test levels. ***, **, * indicates significance at the 1%, 5%, 10% levels.

**Table 11:** Forecasting performance of VECM model.

	Training Data Set	Testing Data Set
RMSE	1.206	1.074
MAE	1.023	0.813
MAPE	18.212	20.538
MASE	-	-

**Table 12:** Evaluation statistics for test data (2018Q3–2022Q4).

Combination Tests			
Null Hypothesis: Forecast i Includes All Information Contained in Others			
**Forecast**	**F-Stat**	**F-Prob**	
ARFIMA	14.59972	0.0015	
SETAR	11.96870	0.0029	
VECM	3.188340	0.0898	
**Evaluation Statistics**			
**Forecast**	**RMSE**	**MAE**	**MAPE**
ARFIMA	1.003651	0.830556	16.66006
SETAR	1.010153	0.720912	13.61785
VECM	1.074261	0.813869	15.74469
Simple mean	1.021395	0.779186	15.12554

**Table 13:** The empirical results of Diebold–Mariano test (HLN adjusted) for test set.

	Accuracy	Statistic	<Prob
ARFIMA vs. SETAR	Abs Error	1.525568	0.0777
VECM vs. SETAR	Abs Error	−4.682692	0.9997
ARFIMA vs. VECM	Abs Error	0.249761	0.4037

## Data Availability

The data source of the series being the dataset of European Union labor force survey, EU-LFS. This data can be found here: [https://ec.europa.eu/eurostat/web/lfs/data/database, une_rt_q, une_rt_m] (accessed on 11 August 2021).
